# RNA Binding Proteins and Gene Expression Regulation in *Trypanosoma cruzi*

**DOI:** 10.3389/fcimb.2020.00056

**Published:** 2020-02-20

**Authors:** Bruno A. A. Romagnoli, Fabiola B. Holetz, Lysangela R. Alves, Samuel Goldenberg

**Affiliations:** Gene Expression Regulation Laboratory, Institute Carlos Chagas, Curitiba, Brazil

**Keywords:** *Trypanosoma cruzi*, gene expression regulation, RNA binding proteins, CRISPR/CAS, zinc finger protein, RNA granules

## Abstract

The regulation of gene expression in trypanosomatids occurs mainly at the post-transcriptional level. In the case of *Trypanosoma cruzi*, the characterization of messenger ribonucleoprotein (mRNP) particles has allowed the identification of several classes of RNA binding proteins (RBPs), as well as non-canonical RBPs, associated with mRNA molecules. The protein composition of the mRNPs as well as the localization and functionality of the mRNAs depend on their associated proteins. mRNPs can also be organized into larger complexes forming RNA granules, which function as stress granules or P-bodies depending on the associated proteins. The fate of mRNAs in the cell, and consequently the genes expressed, depends on the set of proteins associated with the messenger molecule. These proteins allow the coordinated expression of mRNAs encoding proteins that are related in function, resulting in the formation of post-transcriptional operons. However, the puzzle posed by the combinatorial association of sets of RBPs with mRNAs and how this relates to the expressed genes remain to be elucidated. One important tool in this endeavor is the use of the CRISPR/CAS system to delete genes encoding RBPs, allowing the evaluation of their effect on the formation of mRNP complexes and associated mRNAs in the different compartments of the translation machinery. Accordingly, we recently established this methodology for *T. cruzi* and deleted the genes encoding RBPs containing zinc finger domains. In this manuscript, we will discuss the data obtained and the potential of the CRISPR/CAS methodology to unveil the role of RBPs in *T. cruzi* gene expression regulation.

## Introduction

*Trypanosoma cruzi*, as well as other trypanosomatids, displays several biological features that makes it unique in nature (De Souza, 1984; Rodrigues et al., 2014). One of the most striking features of organisms in the Kinetoplastida order is related to the transcription process, since mRNAs are transcribed as polycistronic units that are later processed to give rise to mature mRNAs (De Gaudenzi et al., 2011; Goldenberg and Avila, 2011). There are some special points concerning this process that should be highlighted. Although RNA polymerase II (RNA pol II) is involved in mRNA transcription, no canonical RNA polymerase II promoters have been identified in trypanosomatids (Clayton, 2016); the mRNAs within a given polycistron are not related in function or in temporal expression during the life cycle of the cells. Additionally, primary transcripts, with a few exceptions, are intronless. The mRNAs are processed by the mechanism of trans-splicing: a common 5′-leader sequence of ~50 nucleotides (varying in size and sequence according to the species) is added to each mRNA within the polycistronic unit, concomitantly with the addition of a 3′ poly-A tail (Palenchar and Bellofatto, 2006; Preußer et al., 2012). Some major questions have been raised concerning the points described above: How are the mRNAs selected for transport to the cytoplasm? How are stage-specific mRNAs selected for translation? To date, definitive answers to these questions are not available, but there are some clues regarding the key players in these processes, hence paving the way to unveiling the mechanisms involved in gene expression regulation in trypanosomes.

In the primitive RNA world, it is conceivable that RNAs existed as naked molecules. However, in all cells, RNAs are covered with proteins and exist as ribonucleoprotein complexes. The proteins associated with RNAs are named RNA-binding proteins (RBPs). There are different classes of RBPs based on the motifs that constitute the RNA binding domains (RBDs). The most common domains, such as the RNA recognition motif (RRM) and zinc finger (ZF) domain, will be discussed below. In addition to the canonical RBPs, there are several proteins involved in metabolism or the stress response that are also associated with RNA and are generally named unconventional RNA binding proteins. It is estimated that 3–10% of a given genome codes for RBPs, corroborating their important role in cell function (Glisovic et al., 2008).

**Figure 1:**
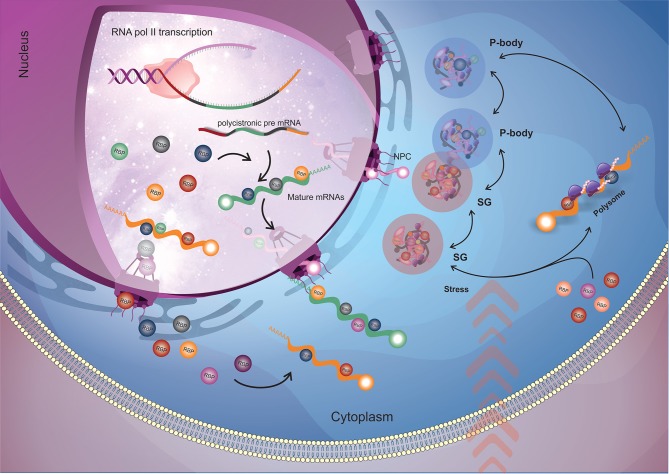
General flow of RBPs action on the mRNA metabolism. Nuclear RBPs interact with their target mRNAs as soon as they start to be transcribed by RNA polymerase II (RNA pol II) and modulate mRNA maturation (trans-splicing and polyadenylation) and nuclear exportation processes. When the mRNAs arrive into the cytoplasm, cytoplasmatic RBPs (along with the ones that came from nucleus) engage their targets and (in a combinatorial signaling) determine the mRNAs fate by directing them to the translational machinery or to RNP granules for storage or degradation. The dynamic destination and trade of mRNAs between those fates relies according to the cellular condition. When submitted to stress (e.g., nutrients availability, pH, temperature), stress granules appear, and in order to recover cell homeostasis, RBPs play a major role in rearranging RNA granules composition by dynamically changing the mRNAs that will address to translation machinery, storage in stress granules or degraded in P-bodies. RBP, RNA binding protein (round shapes); NPC, nuclear pore complex; SG, stress granules; P-body, processing bodies.

RBPs participate in several biological processes, from RNA transcription to decay (Figure 1). In the course of transcription, RNA is wrapped up by RBPs that, in addition to protecting RNA from degradation, play a crucial role in RNA metabolism and fate in the cell. The processing of RNAs (splicing, alternative splicing or trans-splicing) depends on the correct recognition and exposure of RNA sequences to the splicing machinery (Lee and Rio, 2015). After processing, the mRNAs must be transported to the cytoplasm (Björk and Wieslander, 2017) and, once there, the mature mRNAs can be sequestered to the translation machinery for protein synthesis or stored in RNA granules, where they will be kept silent or targeted for degradation. RNA processing, transport within the cell and localization are mediated by RBPs that determine the fate of the mRNA according to their composition in a given mRNP complex (Gerstberger et al., 2014; Re et al., 2014).

The arrangement and assembly of RBPs in mRNP complexes is very dynamic (Müller-McNicoll and Neugebauer, 2013; Ross Buchan, 2014). Different sets of RBPs associate with the mRNA to provide its functionality. The combinatorial arrangement of proteins onto a given mRNP complex has yet to be elucidated and is undoubtedly a great challenge for researchers. It is possible that in addition to the genetic and the histone code, there exists an RBP code. Considering the number of known RBPs and moonlighting proteins with RBP functions, this code is complex and would ultimately determine the genes to be expressed.

The study and characterization of RBPs have greatly increased and improved recently with the development of modern tools that allow interactome studies (Castello et al., 2015; Smirnov et al., 2017). High-throughput techniques, such as mass spectrometry and NGS associated with immunoprecipitation have allowed the isolation and characterization of RBP complexes. This has enabled confirmation of the existence of unconventional RBPs in addition to those that exhibit characteristic features.

## mRNA Decay and Translation

Control of mRNA degradation and access to the translation machinery are very important post-transcriptional processes in *T. cruzi* gene expression regulation. mRNA decay and translation are closely related and can influence each other according to the specific binding proteins associated with the mRNA molecule.

In eukaryotes, the decay of most mRNAs is initiated by the removal of the poly(A) tail. The major mRNA decay pathway of many eukaryotes then proceeds by decapping (by the Dcp1/Dcp2 complex), followed by 5′-3′ exonucleolytic degradation by the exoribonuclease Xrn1 (Parker and Song, 2004).

In trypanosomatids, the 5′-3′ mRNA decay pathway is currently unknown, although deadenylation and mRNA cleavage processes have been extensively studied and appear to be conventional (Erben et al., 2013; Fadda et al., 2014). Removal of the mRNA cap has already been demonstrated *in vitro* (Milone, 2002), but these parasites seem to have developed a decapping mechanism that is different from the Dcp2/Dcp1-mediated decapping found in all other eukaryotes. In fact, the cap structure of trypanosomes is highly unusual: the m^7^GTP residue is followed by four methylated nucleotides forming the cap4 structure (Perry et al., 1987), justifying the need for a distinct decapping enzyme. Recently, Kramer and colleagues (Fritz et al., 2015) standardized a method for the isolation and purification of stress granules in *T. brucei*. From proteomic analyses of these granules, Kramer identified the TbALPH1 protein as the long-sought trypanosome decapping enzyme from trypanosomatids (Kramer, 2017). Depletion of TbALPH1 is lethal and results in a massive, global increase in mRNAs that are deadenylated but have not yet being degraded. This protein has all the characteristics of a decapping enzyme and is colocalized with the TbXRNA protein in the posterior pole granule. Although this posterior pole granule contains two enzymes that are involved directly in mRNAs decay, no mRNA decay intermediates have been found, raising the hypothesis that this granule maintains the separation of degradation enzymes from the pool of mRNAs in the cytoplasm, regulating mRNA decay in a global way. Such a tight, global regulation of mRNA decay is critical for life-cycle progression.

All trypanosomatid digenetic parasites possess non-proliferative life-cycle stages, which are essential for progression between insect and mammalian host. It is known that transcription and translation are downregulated in these stages (Elias et al., 2001; Tonelli et al., 2011), but the exact mechanisms that regulate these processes remain unknown.

Protein biosynthesis is considered a critical step and the ultimate goal for gene expression regulation in trypanosomatids. Recently, Smircich et al. (2015) assessed the extent of regulation of the transcriptome and the translatome in both the non-infective (epimastigote) and infective (metacyclic trypomastigote) forms of *T. cruzi* using RNA-Seq and ribosome profiling methods. The authors showed that a large subset of genes is modulated at the translation level between these two different developmental stages of *T. cruzi*, indicating a key role of translation control during differentiation into the infective form.

Translation initiation is a major contributing step to gene expression regulation and involves several translation initiation factors (eIFs). In eukaryotes, translation initiates with the binding of the tripartite mRNA-binding complex eIF4F (formed by the translation initiation factors eIF4E, eIF4A, and eIF4G) to the cap present at the 5′ end of the mRNAs (Jagus et al., 2012), enabling the attachment of the 43S complex and the two ribosomal subunits to the initiation codon. eIF4E binds directly to the cap and represents a central control point in translational regulation (Jackson et al., 2010). eIF4G, a large scaffold protein, interacts with eIF4E, eIF4A, and PABP. eIF4A, a DEAD-box RNA helicase, unwinds the 5′ proximal region of mRNA. Trypanosomatids possess a large and unusual number of eIF4F translation initiation factor paralogs relative to mammals cells (Freire et al., 2017). There are six eIF4E (eIF4E1-6), five eIF4G (eIF4G1-5), and two eIF4A (eIF4A1-2) homologs. Moreover, trypanosomes have two PABPs (PABP1-2) and *Leishmania* has an additional PABP paralog (PABP3) (da Costa Lima et al., 2010).

The interactions between the different translation initiation factors that form the eIF4F complex have already been determined in *T. brucei* and *Leishmania*, and there are at least five distinct eIF4F (-like) complexes: eIF4E3:eIF4G4; eIF4E4:eIF4G3:PABP1; eIF4E5:eIF4G1; eIF4E5:eIF4G2; and eIF4E6:eIF4G2. It is currently accepted that eIF4E4:eIF4G3:PABP1 is the major translation initiation complex (Freire et al., 2017). However, the exact role of distinct eIF4F-like complexes has not yet been determined, although it is believed that they may be involved in the differential selection of mRNAs. Despite extensive studies on these multiple homologs in *T. brucei* and *Leishmania*, little is known about these factors in *T. cruzi*, and no specific function for each homolog has been characterized. Nevertheless, we have performed a two-hybrid assay comprising screening for all eIF4Es, eIF4Gs, and PABPs from *T. cruzi*. We have identified and confirmed three new interactions: TceIF4E3:TcPABP1, TceIF4E3:TcPABP2, and TceIF4E5:TceIF4G5 (unpublished data). These results strongly suggest a distinct mechanism regarding the translational control of *T. cruzi*.

## RNA Granules

In mammalian and yeast cells, mRNAs that are not being translated, or those destined for degradation, are compartmentalized into distinct cytoplasmic structures generally termed “RNA Granules” or “mRNP Granules” (messenger ribonucleoprotein granules). These granules can be classified in Processing bodies (P-bodies) and stress granules (SG), depending on the presence of specific proteins, and play key roles in the post-transcriptional regulation of gene expression. Stress granules are defined by the presence of translation initiation factors and are involved in the sorting and storage of mRNAs, whereas P-bodies are sites of storage and/or degradation of several transcripts formed by the presence of translation-repressor proteins and components of the mRNA degradation machinery (Sheth and Parker, 2003; Teixeira et al., 2005; Holetz et al., 2007; Anderson and Kedersha, 2008; Kedersha and Anderson, 2009). In addition, eukaryotic studies demonstrate that these structures can interact and exchange their components in a dynamic cycle with the exchange of mRNAs between translation, storage and degradation, indicating the decisive role of these structures in the control of gene expression at the posttranscriptional level (Decker and Parker, 2012).

Our previous work pioneered the identification of RNA granules with similarities to P-bodies in *T. cruzi* (Holetz et al., 2007, 2010). Since then, several studies have described the presence of different mRNP granules in *T. cruzi* and *T. brucei*. There are at least six types of mRNP granules in trypanosomatids, namely, P-body-like RNA granules, nutritional stress-induced RNA granules, heat shock-induced RNA granules, nuclear peripheral granules, posterior pole granule, and granules formed by tRNAs (Kramer, 2014). This vast repertoire of mRNP granules can be justified as an adaptation to the loss of transcriptional control. However, despite the importance of translational control and control of mRNA stability in the regulation of gene expression in trypanosomes, the connection between mRNP granules and life cycle regulation remains unknown in these parasites.

Proteins known to be involved in the formation of RNA granules in eukaryotes have been characterized in *T. cruzi*. TcDHH1 is present as a free protein or in polysome-independent complexes localized in *foci* that vary in number and size in response to nutritional stress and to cycloheximide/puromycin treatments, indicating that these structures are in equilibrium with the translation machinery (Cassola et al., 2007; Holetz et al., 2007). Furthermore, TcDHH1 associates with developmentally regulated mRNAs. Accordingly, mRNAs associated with TcDHH1 in the epimastigote stage are those mainly expressed in the other forms of the *T. cruzi* life cycle (Holetz et al., 2010). Interestingly, Dallagiovanna et al. (2008) demonstrated the association of TcPUF6 with TcDHH1 in epimastigote forms but not in metacyclic trypomastigotes. Since TcPUF6 promotes the degradation of its target mRNAs in epimastigotes, it is likely that TcPUF6 regulates the degradation of its associated transcripts by its association with TcDHH1-containing complexes involved in mRNA degradation.

Recently, TcXRNA, the trypanosome Xrn1 homologous protein, was characterized in *T. cruzi* (Costa et al., 2018a). TcXRNA exhibits granular cytoplasmic cell localization, is constitutively expressed throughout the life cycle of *T. cruzi* and accumulates at the nuclear periphery when mRNA processing is inhibited. TcXRNA does not colocalize with TcDHH1 and TcCAF1 (a catalytic subunit of the Ccr4-Not deadenylase complex) granules in the cytoplasm. On the other hand, the colocalization of TcXRNA with distinct mRNP granules occurs mainly around the nucleus, which suggests the existence of an mRNA quality control checkpoint at the nuclear periphery, involving the activity of distinct proteins, such as TcXRNA, TcDHH1, and TcCAF1.

The precise function of mRNP granules as well as their relationship with translational control and life-cycle regulation remain poorly understood in *T. cruzi*. The data obtained so far indicate the existence of several types of granules formed as a result of different stimuli, whose assembly is dependent on mRNAs. Furthermore, although they can share several proteins, there are distinct structures that interact with each other dynamically. The mechanism that distinguishes mRNAs destined for storage or degradation in *T. cruzi* seems to depend on the combination of different protein components associated with the mRNAs, according to their expression levels during the life cycle, corroborating the complexity of the process of regulation of gene expression in this parasite.

## RNA-Binding Proteins in *Trypanosoma cruzi*

### RBPs With RRM Domains in *T. cruzi*

The RNA-recognition motif (RRM) is the most common and versatile domain found in RBPs as it can bind different molecules, such as single- and double- stranded RNA and DNA and interact with proteins. Due to its plasticity, RBPs that contain this domain are key players in RNA metabolism, acting from mRNA splicing to mRNA turnover (Cléry et al., 2008; Cléry and Frédéric, 2012). A summary of the RBPs studied in *T. cruzi* are described in Table 1.

**Table 1:** List of RBPs characterized in *T. cruzi*.

**RBP name**	**RBP domain**	**Stage expression**				**Regulated throughout life cycle**	**Localization**	**References**
		**A**	**E**	**M**	**T**			
TcAlba 30	ALBA	•	•	•	•	No	Cytoplasmatic	Pérez-Díaz et al., 2017
EF-1α	Non cannonical	•	•	•	•	Yes	Cytoplasmatic	Alves et al., 2015
TcPIWI-tryp	PIWI/OB-fold	•	•	•	•	No	Cytoplasmatic	Garcia Silva et al., 2010; Garcia-Silva et al., 2014
TcPUF1	PUF	–	•	–	–	Not reported	Cytoplasmatic	Caro et al., 2006
TcPUF6	PUF	•	•	•	•	No	Cytoplasmatic	Dallagiovanna et al., 2005, 2008
PABP1	RRM	•	•	•	•	No	Cytoplasmatic	Batista et al., 1994
TcDRBD2	RRM	•	•	•	•	No	Cytoplasmatic	Wippel et al., 2019
TcDRBD4/PTB2	RRM	Nr	•	Nr	Nr	Not reported	Cytoplasmatic /Nuclear	Jäger et al., 2007; De Gaudenzi et al., 2016
TcNrBD1	RRM	•	•	•	•	No	Cytoplasmatic	Oliveira et al., 2016
TcRBP3	RRM	–	•	–	–	Yes	Cytoplasmatic	De Gaudenzi et al., 2003
TcRBP4	RRM	–	•	–	–	Yes	Cytoplasmatic	De Gaudenzi et al., 2003
TcRBP5	RRM	•	•	•	•	Yes	Cytoplasmatic	De Gaudenzi et al., 2003
TcRBP6	RRM	•	•	•	•	Not reported	Cytoplasmatic	De Gaudenzi et al., 2003
TcRBP9	RRM	Nr	•	–	Nr	Yes	Cytoplasmatic	Wippel et al., 2018a
TcRBP19	RRM	•	–	–	–	Yes	Cytoplasmatic	Pérez-Díaz et al., 2007, 2012, 2013
TcRBP40	RRM	•	•	–	•	Yes	Cytoplasmatic ^*^	Guerra-Slompo et al., 2012
TcRBP42	RRM	•	•	•	•	Not reported	Cytoplasmatic	Tyler Weisbarth et al., 2018
TcRBSR1	RRM	•	•	–	•	Yes	Cytoplasmatic /Nuclear	Wippel et al., 2018b
TcTRRM1/TcSR62	RRM/ZF(C2HC)	Nr	•	Nr	Nr	Not reported	Nuclear	Názer et al., 2011; Wippel et al., 2018b
TcUBP1	RRM	•	•	•	•	Yes	Cytoplasmatic	D'Orso and Frasch, 2002
TcUBP2	RRM	•	•	–	–	Yes	Cytoplasmatic	D'Orso and Frasch, 2002
TcZC3H29	ZF(C3H)	–	•	–	–	Yes	Cytoplasmatic	This work
TcZC3H31	ZF(C3H)	•	•	•	•	Yes	Cytoplasmatic	Alcantara et al., 2018
TcZC3H39	ZF(C3H)	•	•	•	•	No	Cytoplasmatic	Alves et al., 2014
TcZC3HTTP	ZF(C3H)	–	•	–	–	Yes	Cytoplasmatic	This work
TcZFP1	ZF(C3H)	•	•	•	•	Yes	Not reported	Mörking et al., 2004
TcZFP2	ZF(C3H)	•	•	•	•	Yes	Cytoplasmatic	Mörking et al., 2012
TcZFP8	ZF(C3H)	•	•	•	•	No	Nuclear	Ericsson et al., 2006

* TcRBP40 localizes in reservosomes in epimastigotes forms.

Nr, Not reported.

*Other RBPs investigated in T. cruzi but without further characterization are TcPUF3, TcPUF5, TcPUF8 (Caro et al., 2006) and TcRBP10 (Wippel et al., 2018a)*.

In *T. cruzi*, most of the RBPs characterized to date present RRM domains and are involved in RNA metabolism by controlling the transcripts stability and turnover. One well-characterized RBP is TcUBP1, which presents a single RRM domain and form both stabilizing or destabilizing interactions depending on its partners in the mRNP complex (D'Orso and Frasch, 2002; Volpon et al., 2005; Li et al., 2012; Sabalette et al., 2019). The protein recognizes the AU-rich elements located at the 3′-untranslated region (UTR) of mucin *SMUGL* mRNAs (D'Orso and Frasch, 2002; Li et al., 2012). In addition, it has been recently shown that overexpression of TcUBP1 in epimastigotes increases by 10-fold the amount of transcripts coding for surface proteins and that they were being actively translated (Sabalette et al., 2019). The ectopic expression of TcUBP1 in trypomastigotes increased the infectivity rates, demonstrating the important role of this protein for the parasite virulence (Sabalette et al., 2019). TcUBP2 is another RRM-containing protein and is part of the TcUBP1 complex. It acts by binding to the poly(U) region of the *SMUGL* mucin mRNA that acts to control the expression of this transcript (D'Orso and Frasch, 2002).

TcDRBD4/PTB2 is an RBP presenting two RRM domains that play a role in the destabilization of the *ubp1* and *ubp2* mRNAs. It regulates splicing and prevents trans-splicing by binding in the regulatory elements present in the intercistronic region (ICR) of the *ubp1* and *ubp2* genes. These results indicate that TcDRBD4/PTB2 might act by covering the trans-splicing/polyadenylation signals (De Gaudenzi et al., 2016).

TcRBP19 presents a single RRM domain, whose ectopic overexpression impairs the parasite's life cycle and infection ability. This RBP led to a reduction in the number of infected cells (Pérez-Díaz et al., 2012). TcRBP19 presents a low level of expression in the epimastigote forms of the parasite. It was shown that this protein binds to the 3′-UTR region of its own transcript, decreasing its stability and suggesting its role as a destabilizing factor (Pérez-Díaz et al., 2013).

TcRBP40 also presents a single RRM domain. It binds to AG-rich regions in the 3′-UTR of target mRNAs coding for transmembrane proteins. In addition, the TcRBP40 protein is localized in reservosomes in the replicative epimastigote form; this organelle is associated with protein and lipid storage. In the mammalian-host forms of the parasite, amastigotes and trypomastigotes, it is diffused in the cytoplasm. This suggests a regulatory function for this protein due to its shift in cellular localization according to the developmental stage of the parasite (Guerra-Slompo et al., 2012).

TcRBP9 is a cytoplasmic protein that presents one RRM domain. The protein is associated with translational complexes, suggesting its involvement in translation regulation. RBP9 associates with other RBPs involved in RNA metabolism, such as ZC3H39, UBP1/2, NRBD1, and ALBA3/4. When parasites under stress were analyzed, irrespective of RBPs, the translation initiation factors eIF4E5, eIF4G5, eIF4G1, and eIF4G4 were also identified. In addition, the RBP9-mRNP complex regulates transcripts coding other RBPs, such as RBP5, RBP6, and RBP10 and proteins involved in metabolic processes. These results indicate that RBP9 is part of a cytoplasmic mRNP complex involved in mRNA metabolism and translation regulation (Wippel et al., 2018a).

TcNRBD1 is an RBP that contains two RRM domains and is expressed throughout the life cycle of *T. cruzi*. This protein is orthologous to the P34 and P37 proteins from *T. brucei*, although the role they play in these organisms is distinct. TcNRBD1 is localized at the perinuclear region and associates with either 80S ribosomes or polysomes, indicating its role in the translation process. This observation was corroborated by ribonomic analysis that showed several transcripts encoding ribosomal proteins associated with TcNRBD1. Proteomic analysis also indicated that TcNRBD1 associates with several ribosomal proteins from both the 40S and 60S subunits, reinforcing its role in the translation process (Oliveira et al., 2016).

TcRBSR1 is a predominantly nuclear RBP that contains one RRM domain and a serine-arginine (SR)-rich region; this protein seems to be developmentally regulated since no expression is detected in the infective metacyclic trypomastigote forms. Proteomic data showed that TcRBSR1 interacts with other RBPs, such as TcUBP1, TcUBP2, and TcTRRM1. An immunoprecipitation assay followed by RNA-seq indicated that RBSR1-mRNP binds to snoRNAs and snRNAs, leading to a hypothesis regarding its role in RNA processing in the nucleus (Wippel et al., 2018b).

TcRBP42 is a cytoplasmic RBP that presents one RRM domain and one NTF2-like domain. The NTF2 domain is associated with nuclear-cytoplasmic transport (Aibara et al., 2015). RBP42 is expressed in all developmental forms of *T. cruzi*, suggesting a role in gene expression regulation throughout the life cycle of the parasite. It was shown that overexpression of the protein did not lead to any alteration in the capacity of *T. cruzi* to differentiate into the metacyclic trypomastigote form or in cell infection capacity, as previously described for its ortholog in *T. brucei* (Tyler Weisbarth et al., 2018).

### RBPs With the CCCH Zinc Finger Domain in *T. cruzi*

Zinc finger proteins (ZFP) were originally identified as DNA binding proteins with a molecular arrangement of two cysteine and two histidine residues that coordinate a zinc ion. However, it was later demonstrated that a class of zinc finger proteins characterized by the presence of the domain Cys–Cys–Cys–His (CCCH)- binds to RNA molecules (Hall, 2005).

In *T. cruzi*, the ZFP protein TcZFP1 presents a C(2)H(2) domain and specifically binds cytosine-rich repetitive sequences *in vitro* present in untranslated regions of many mRNAs in trypanosomatids (Mörking et al., 2004). TcZFP2 is also a C(2)H(2) ZFP that binds transcripts associated with parasite-host interactions. It was shown that the mRNAs bound to this protein are downregulated in the replicative forms, indicating that the TcZFP2 protein might act as a destabilizing factor (Mörking et al., 2012). In addition, TcZFP1 and TcZFP2 interact with each other via WW domain in TcZFP2A (Caro et al., 2005). TcZFP8 is a zinc finger protein that presents a nuclear localization that might act in RNA metabolism in *T. cruzi* nucleus (Ericsson et al., 2006).

The ZFP protein TcZC3H39 presents a CCCH domain and a U-box domain. The U-box domain is involved in substrate specificity for ubiquitination (Christensen and Klevit, 2009). TcZC3H39 is associated with the stress response in *T. cruzi*: it binds to highly expressed mRNAs that code for cytochrome c oxidase (COX) enzymes and ribosomal proteins, slowing their translation under stress conditions. Interestingly, TcZC3H39 associates with transcripts that are related in function, hence providing support to the RNA regulon theory (Alves et al., 2014).

TcZC3H31 is a cytoplasmic CCCH ZFP expressed in epimastigotes and metacyclic trypomastigotes. Deletion of *zc3h31* led to the impairment of epimastigote differentiation into the metacyclic trypomastigote form. In addition, when insects were infected with *zc3h31* KO cells, the parasites presented an altered morphology relative to wild-type cells, indicating a delay in differentiation. Moreover, in cells overexpressing TcZC3H31, the differentiation rate from epimastigotes into metacyclic trypomastigotes was more efficient than that in wild-type epimastigotes. These results indicate that this ZFP is an important cell cycle regulator in *T. cruzi* (Alcantara et al., 2018).

### Other RBP Domains in *T. cruzi*

The PUF (Pumilio/Fem-3 mRNA binding factor) protein family of RBPs is very common in higher eukaryotes; these proteins recognize cis-elements in the 3′-UTR of the mRNAs, regulating their stability and function. In *T. cruzi*, there are eight putative PUF proteins annotated in the genome (Caro et al., 2006); the protein TcPUF6 was characterized in epimastigotes and demonstrated to be involved in the destabilization of specific mRNAs that are upregulated in the infective trypomastigote forms of the parasite (Dallagiovanna et al., 2008).

TcSR62 belongs to the family of serine/arginine (SR)-rich proteins; it is a cytoplasmic RBP implicated in the stress response in *T. cruzi* upon actinomycin D (ActD) treatment. TcSR62 relocates to the nucleolus when transcription is inhibited in epimastigotes along with other RBPs, specifically PTB (polypyrimidine tract-binding protein) and PABP1 (poly A binding protein 1). Interestingly, the same pattern of nucleolar localization was observed with poly(A+) mRNAs after ActD treatment. Altogether, these results suggest that the nucleolus could play a role in accumulating and protecting mRNAs and associated RBPs when cells are subjected to a specific stress condition (Názer et al., 2011).

The canonical RNAi pathway is not functional in *T. cruzi*; however, a canonical Argonaute (AGO/PIWI), named TcPIWI-tryp, is present and expressed throughout the life cycle of the parasite (Garcia Silva et al., 2010). Sequencing of TcPIWI-tryp-associated RNAs showed enrichment for small RNAs, mainly derived from rRNAs and tRNAs. The composition of small RNAs in the *T. cruzi* TcPiWI/AGO protein is distinct from those identified in other eukaryotes, suggesting that in this parasite, the protein might present distinct biological functions (Garcia-Silva et al., 2014).

Members of the Alba (acetylation lowers binding affinity) protein family bind DNA and interact with distinct RNA molecules and form mRNP complexes. TcAlba30 is an Alba protein in *T. cruzi* that is expressed in all stages of the parasite life cycle. Ribonomic analysis showed that TcAlba30 can interact with β-amastin mRNA. When the protein was overexpressed, the levels of β-amastin decreased by 50%, indicating a role in the negative control of β-amastin expression (Pérez-Díaz et al., 2017).

There is much evidence showing that proteins without canonical RNA-binding domains (RBDs) can interact with RNA molecules; these are known as moonlighting proteins, and they have been extensively studied (Collingridge et al., 2010; Huberts et al., 2010; Lindner et al., 2013; Müller-McNicoll and Neugebauer, 2013; Gil-Bona et al., 2015). One example of an RNA binding protein lacking RBD in *T. cruzi* is elongation factor 1 α (EF-1α), which plays a canonical role in translation. This protein responds to stress conditions by binding a specific subset of mRNAs. The associated mRNAs showed enrichment of gene sets involved in single-organism metabolic processes, amino acid metabolic processes, ATP and metal ion binding and glycolysis. EF-1α co-sedimented with heavy complexes that were not associated with the translation machinery, reinforcing the “moonlighting” role of this protein during stress conditions (Alves et al., 2015).

## Knockout of RBP Genes

Despite all the advances regarding the function of RBPs in trypanosomes, the roles of many of these proteins in the parasite's gene regulatory network remain unknown. To address this issue, in addition to the already mentioned high-throughput techniques, other resourceful methodologies are being used to study RBPs. They consist essentially of genetic reverse approaches, such as overexpression and/or gene knockdown/knockout. Indeed, our current knowledge about the roles of individual RBPs came mostly from studies that modulated their endogenous levels by increasing and/or decreasing/abolishing their expression and investigating the impact on the vital processes of the parasites, such as proliferation, differentiation, and infection, among others.

In *T. brucei*, studies involving RBP gene silencing (knockdown) by the interference RNA (RNAi) machinery provided a major contribution to the field and allowed the investigation of many RBP functions (Estévez, 2008; Archer et al., 2009; Ling et al., 2011; Subota et al., 2011; Das et al., 2012; Wurst et al., 2012; Droll et al., 2013; Levy et al., 2015). In *T. cruzi*, however, since the RNAi machinery is not functional, to understand the impact of the absence of a specific RBP, researchers had to attempt gene knockout by incorporating a DNA cassette containing a selective drug marker into a target gene by homologous recombination. However, due to the limitations of this methodology, only one group reported success in knocking out RBPs with this approach (Alcantara et al., 2018). Accordingly, this approach is not feasible in cases where the target gene is essential, which seems to be the case for many RBPs, as reported in *T. brucei* through gene silencing assays (Das et al., 2012; Wurst et al., 2012; Droll et al., 2013; Fernández-Moya et al., 2014; Jha et al., 2015; Levy et al., 2015).

Therefore, in order to further advance comprehension of this important set of regulatory proteins in *T. cruzi*, new and more efficient genetic editing technologies are required. Fortunately, the CRISPR/Cas9 DNA editing system has recently emerged as a resourceful and promising tool for reverse genetics approaches and has already been adapted for several organisms, including those that are considered challenging to manipulate genetically, as is the case with *T. cruzi* (Lander et al., 2015, 2016; Peng et al., 2015; Soares Medeiros et al., 2017; Costa et al., 2018b; Romagnoli et al., 2018). Recently, we proposed some modifications to previous CRISPR/Cas9 (Lander et al., 2015; Peng et al., 2015) methods for knockout generation in *T. cruzi* (Romagnoli et al., 2018). Our goal was to establish a protocol with maximum disruption efficiency and a way to investigate/confirm related phenotypes as quickly as possible, which is crucial considering that disruption of important regulatory elements is likely to be essential to the parasite. Briefly, our current strategy consists of generating a highly enriched and stable population expressing Cas9-GFP and then transfecting this population with desired *in vitro*-produced guide RNAs (gRNAs) to target RBP genes along with a repair single-stranded DNA template containing a unique restriction site and a sequence that encodes the stop codons in three different frames. Our initial strategy did not use a DNA donor for direct double-strand break repair (Romagnoli et al., 2018). However, based on other reports that highlighted an increase in gene disruption efficiency and specificity in the presence of a single-strand DNA donor (Lander et al., 2015; Zhang et al., 2017; Burle-Caldas et al., 2018), we incorporated the use of a DNA template into our strategy as well.

To date, a few reports with slightly different application strategies have used the CRISPR/Cas9 system to manage gene disruption in *T. cruzi*, but as yet, none have reported its use to knock out and study RBP genes. The knockout of RBP genes in *T. cruzi* should provide new clues about the role of RBPs in gene expression regulation of the parasite. We will show and discuss below a CRISPR/Cas9 approach to perform the knockout of RBPs in *T. cruzi*, using as examples some of the RBPs that we are investigating.

**Figure 2:**
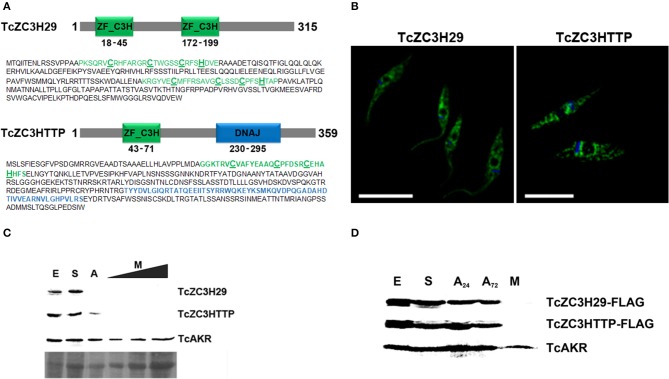
The *T. cruzi* zinc finger proteins TcZC3H29 and TcZC3HTTP. **(A)** Graphic representation and amino acid sequences of the proteins. The zinc finger C3H domains are highlighted in green (with the cysteine and histidine residues underlined in bold) and the TcZC3HTTP DNAJ domain in blue. **(B)** Immunolocalization of TcZC3H29 and TcZC3HTTP. Epimastigotes were incubated with anti-TcZC3H29 (1:300) or anti-TcZC3HTTP (1:750), and an Alexa 488-conjugated goat anti-mouse antibody (1:600) was used for detection. The nucleus and kinetoplast are stained with DAPI. Bar = 10 μm. **(C)** TcZC3H29 and TcZC3HTTP expression profiles during metacyclogenesis. Western blot of protein extracts obtained from 5 × 10^6^ parasites in distinct differentiation stages. Epimastigotes (E), epimastigotes after 2 h of nutritional stress (S), nutritionally stressed epimastigotes in the adhesion stage (A) and metacyclic trypomastigotes (M). To further investigate the expression of TcZC3H29 and TcZC3HTTP in the infective form, increased amounts of metacyclic trypomastigotes extract (5 × 10^6^, 1 × 10^7^, and 1.5 × 10^7^ parasites) were used. Detection was performed with antisera against TcZC3H29 (1:500) and TcZC3HTTP (1:1,000) and anti-TcAKR (1:1,000). TcAKR (Aldo-keto reductase, TCDM_00490) was used as a normalizer. A portion of the Ponceau-S stained blot is shown to demonstrate sample input. **(D)** 3xFLAG C-terminally tagged TcZC3H29 and TcZC3HTTP detection during metacyclogenesis. Protein extracts were prepared from 5 × 10^6^ parasites at different metacyclogenesis stages and incubated with anti-FLAG antibodies (1:1,000). A_24_–nutritionally stressed adhered epimastigotes after 24 h; A_72_–nutritionally stressed adhered epimastigotes after 72 h.

In addition to the already mentioned TcZC3H39, we also present data from the knockout assays of two other RBPs, TcZC3H29 (TCDM_11529), and TcZC3HTTP (TCDM_03704). These two zinc finger proteins are unique to Kinetoplastida and contain C3H domains (Kramer et al., 2010). While TcZC3H29 has two C3H domains (C-X_7_-C-X_5_-CX_3_-H and C-X_8_-C-X_4_-C-X_3_-H), TcZC3HTTP has one C3H domain (C-X_8_-C-X_5_-C-X_3_-H), and a DNAJ domain (Figure 2A). Proteins TcZC3H29 and TcZC3HTTP present cytoplasmatic localization with a granular pattern (Figure 2B), similar to that reported for TcZC3H39 (Alves et al., 2014). Our interest in these two particular RBPs was dictated by the fact that they are exclusively expressed in the non-infective epimastigote form (data not shown). Accordingly, they are downregulated in the course of differentiation into infective metacyclic trypomastigotes (Figure 2C). Interestingly, even ectopically overexpressed Flag-tagged TcZC3H29 and TcZC3HTTP were not detected in metacyclic trypomastigotes (Figure 2D), thus indicating the existence of tight regulatory control acting at stage-specific expression levels and pointing their crucial role in *T. cruzi* development.

**Figure 3:**
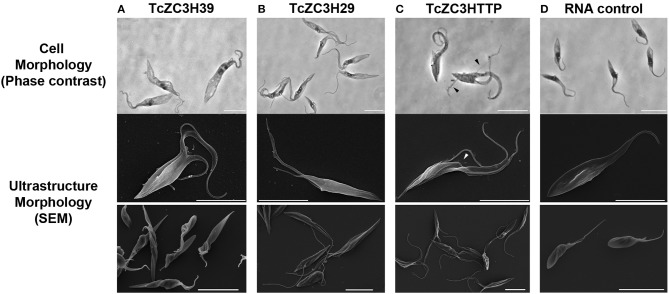
Knockout of TcZC3H39, TcZC3H29, and TcZC3HTTP results in *T. cruzi* morphological changes. Parasites were visualized by light microscopy (upper lane) and scanning electron microscopy in higher or lower magnifications (middle and bottom lanes, respectively) 3 days after transfection with specific gRNAs targeting TcZC3H39 **(A)**, TcZC3H29 **(B)**, or TcZC3HTTP **(C)**. Parasites expressing Cas9-GFP were also transfected with a non-guide RNA (RNA Control) as an experimental control **(D)**. Arrow heads are indicating smaller flagella Bar: 10 μm.

Initial attempts to use the CRISPR/Cas system involved knocking out *T. cruzi* GP72, and the cells presented the typical flagellum detachment phenotype (Lander et al., 2015; Romagnoli et al., 2018). Next, we attempted to knock out TcZC3H39, TcZC3H29, and TcZC3HTTP by performing electroporation with specific guide RNAs to target their respective genes (Supplementary Table 1). After transfection, major morphological changes were observed in the cultures (Figures 3A–C). For TcZC3H39 and TcZC3H29, cells presented a larger size, an extension of the posterior region and more than one flagellum per cell (Figures 3A–B, respectively). The cultures targeted with TcZC3H29 gene disruption presented additional flagella that were thinner and longer than those found in control parasites or when targeting TcZC3H39 or TcZC3HTTP (Figure 3 comparing B–D, A, and C, respectively). The targeting of TcZC3HTTP resulted in larger cells, but they did not have the body extension that was observed for the two other zinc finger RBP knockouts (Figure 3C). In addition, the cell division process of the transfected parasites seemed to be affected, as two fully formed flagella and duplication of the anterior region were frequently observed. Smaller flagella were also observed (Figure 3C). Surprisingly, parasites in final stages of cell division (just before cytokinesis, when cells are connected by their posterior end with one flagellum in each anterior end (Alcantara et al., 2017) were observed with another new flagellum already in each anterior end (Supplementary Figure 1), thus reinforcing the idea that these cells somehow lost at least part of their cell cycle coordination.

**Figure 4:**
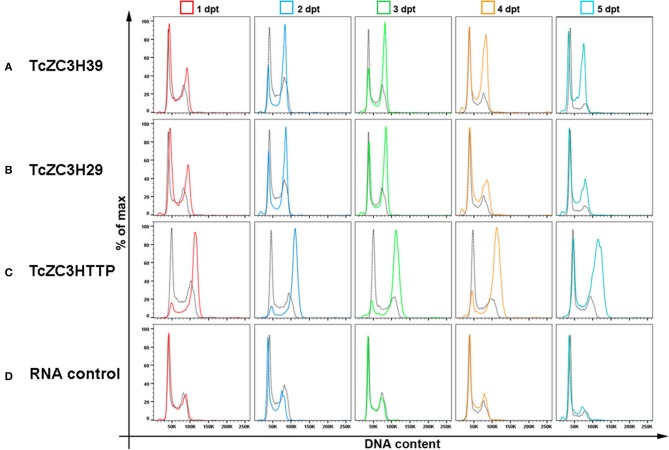
Cell cycle impairment by disruption of the genes encoding TcZC3H39, TcZC3H29 and TcZC3HTTP. Populations transfected with specific gRNAs to **(A)** TcZC3H39, **(B)** TcZC3H29, or **(C)** TcZC3HTTP underwent cell cycle assessment by flow cytometry from day 1 to 5 post transfection (1–5 dpt). Graphs show the percentage of total cells (y-axis) by the amount of DNA (x-axis) in each transfected population line, with specific gRNAs, and on each day of analysis (rows). **(D)** A non-guide RNA (control RNA) was used as the CRISPR/Cas 9 system specificity control. Each graph also shows the wild-type population cell cycle (black dotted) for comparison.

To further investigate the impact of targeting these zinc finger proteins on cell cycle, we analyzed the DNA content of transfected cells by flow cytometry from day 1 to 5 after gRNA transfection. In general, cell cycle kinetic analysis revealed an increase in the number of parasites at the G2/M phase when targeting TcZC3H39, TcZC3H29, or TcZC3HTTP (Figures 4A–C, respectively). The TcZC3H29 gene disruption attempt provoked a significant accumulation of parasites with double DNA content in the first 3 days of the analysis (Figure 4A), whereas TcZC3H39 and TcZC3HTTP presented this phenomenon throughout the entire kinetic analysis (Figures 4B–C). Interestingly, TcZC3HTTP targeting resulted in a significant number of parasites with DNA content slightly above the value considered to be double (Figure 4C). The meaning of this observation remains to be elucidated. Nevertheless, cell cycle analysis indicates that the attempt to knock out any of these zinc finger proteins impaired cell cycle progression, likely due to the inability of the cells to complete the division process, thus raising the possibility that TcZC3H39, TcZC3H29, and TcZC3HTTP are essential genes in *T. cruzi*. Accordingly, all the attempts to clone (or even enrich) and culture the morphologically affected parasites have been unsuccessful (data not shown).

It is important to mention that TcZC3H39, TcZC3H29, and TcZC3HTTP gene disruption was performed at least three times using different designed gRNAs, and the observed effects were reproduced in all attempts with all guide RNAs (Supplementary Figure 2). In addition, the phenotypes observed for the zinc finger targets were not observed in the control Cas9-GFP expressing population, wild-type cells or even the Cas9-GFP population parasites transfected with a control RNA (Supplementary Figure 3). Additionally, these experiments were performed in parallel with the knockout of other known proteins, such as GP72 and α-tubulin, which presented previously described morphological phenotypes [data published elsewhere (Lander et al., 2015; Romagnoli et al., 2018)].

The changes observed in the TcZC3H39, TcZC3H29, and TcZC3HTTP knockout populations are specific and likely due to the absence of these proteins in *T. cruzi*. Western blot analysis to assess the expression of TcZC3H29 and TcZC3HTTP in the transfected populations showed the absence of the proteins only in the cultures electroporated with gRNAs targeting their encoding gene (Supplementary Figure 4). However, as yet we were not able to detect gene editing at the DNA level (data not shown).

**Figure 5:**
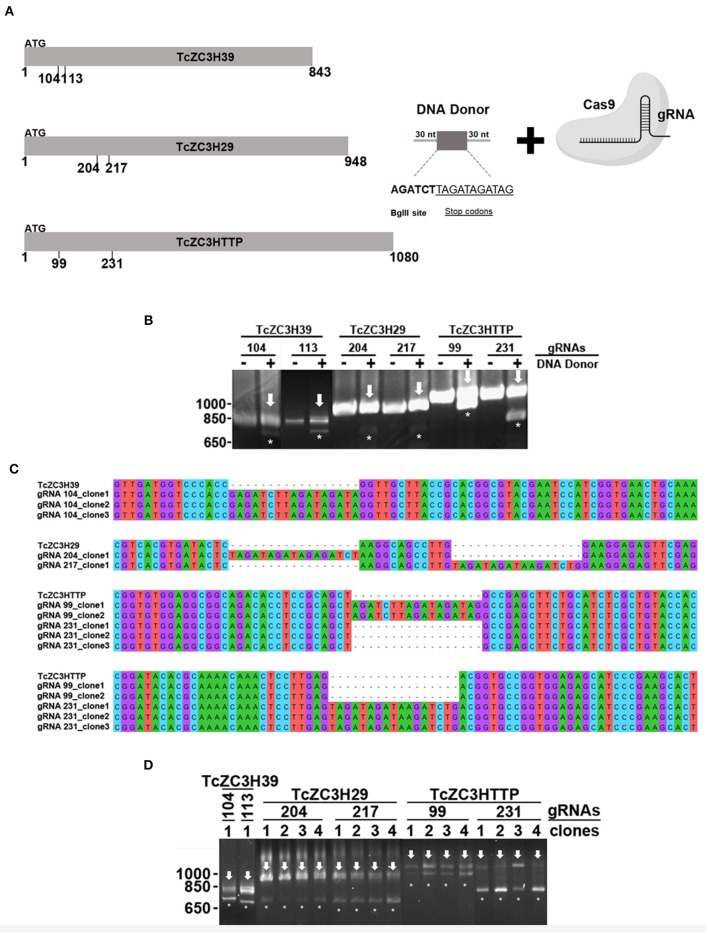
Disruption of the genes encoding TcZC3H39, TcZC3H29, and TcZC3HTTP by CRISPR/Cas9 using a DNA donor strategy. **(A)** Scheme depicting the DNA donor strategy used for gene knockout. Zinc finger genes (represented on the left as TcZC3H39, TcZC3H29, and TcZC3HTTP) were targeted by Cas9 (through specific gRNA recognition) in different regions (the positions are indicated below each gene representation) along with a DNA template containing a 30-nt homology arm (at each end), a *Bgl*II restriction site (AGATCT) and a sequence that encodes stop codons in three different frames (TAGATAGATAG). **(B)** Genomic DNA from transfected populations with gRNAs targeting TcZC3H39, TcZC3H29, or TcZC3HTTP with (+) or without (–) the respective DNA donors was PCR amplified with specific primers for each zinc finger gene and digested with *Bgl*II for gene disruption visualization. Asterisks (*) highlight *Bgl*II restriction site incorporation by showing the digested products, whereas the remaining undigested amplicon is indicated by an arrow. **(C)** DNA sequencing of the tczc3h39, tczc3h29, and tczc3http genes showing correct insertion of the DNA donor. After transfection, zinc finger targeted genes were amplified from genomic DNA, cloned into the pGEM-T Easy vector (Promega) and transformed into the TOP10 chemically competent *Escherichia coli* strain. Then, plasmids were isolated from the positive clones (identified by PCR and *Bgl*II digestion) and sent for sequencing for gene disruption confirmation. **(D)** Confirmation of single cell sorted clones containing the tczc3h39, tczc3h29, or tczc3http disrupted genes. Genomic DNA from the single cell cloned (by flow cytometry) population was used for tczc3h39, tczc3h29, or tczc3http PCR amplification followed by *Bgl*II digestion. At least one clone is representatively shown for each gene according to the specific gRNA used to achieve gene knockout (indicated above).

This concern led us to adapt the experimental approach in order to improve the gene disruption identification capability. Hence, for TcZC3H39, TcZC3H29, and TcZC3HTTP knockouts, in addition to the respective designed guide RNAs to specifically target the genes, a DNA template was designed and included into the transfection process to direct specific gene repair. This DNA template strategy was designed based on previous reports (Zhang and Matlashewski, 2015; Zhang et al., 2017; Burle-Caldas et al., 2018) and consists of a 77-nt single strand oligo DNA donor containing homology arms (30 nt at each end), a restriction site for *Bgl*II, and a sequence that encodes three stop codons in three different frames (Figure 5A). After transfecting the gRNAs along with their corresponding DNA donors (the TcZC3H39, TcZC3H29, and TcZC3HTTP genes), repair/disruption was confirmed by polymerase chain reaction directly from liquid culture (Alcantara et al., 2014). The amplified target gene products were digested with the *Bgl*II enzyme (Figure 5B). As shown, only the cultures co-transfected with the gRNAs and the related single-stranded DNA oligos successfully incorporated the *Bgl*II restriction site at the target gene although the DNA donor incorporation occurred with distinct efficiencies (Supplementary Figure 5). Furthermore, the amplified target genes were cloned into the pGEM-T Easy vector (Promega) and sequenced for correct repair visualization (Figure 5C). However, some of the PCR product remained undigested (Figure 5B, arrows), indicating that in the transfected cultures there were parasites that did not have the target RBP gene disrupted/repaired, or the editing may have occurred in one allele only. To test the hemi-knockout hypothesis, single-cell sorting was performed to individualize the transfected parasites. After growth, clones from all sorted cultures were tested again by PCR and *Bgl*II digestion. In all sorted populations, clones were observed that did not exhibit incorporation of the *Bgl*II restriction site (data not shown), thus pointing to the fact that gene disruption did not achieve 100% efficiency.

**Figure 6:**
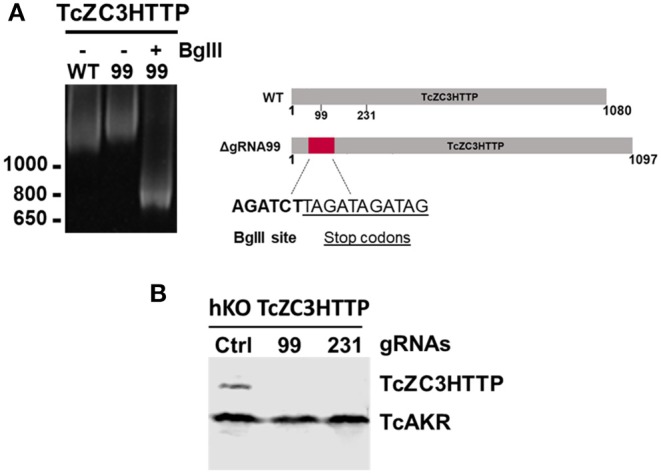
TcZC3HTTP knockout confirmation after transfection with specific gRNAs. **(A)** 5% polyacrylamide gel electrophoresis to confirm DNA donor incorporation in the TcZC3HTTP gene. Genomic DNA from cloned population (by flow cytometry) was used for tczc3http PCR amplification followed by *Bgl*II digestion (left panel). Scheme depicting the expected increase in TcZ3HTTP gene size and *Bgl*II site and stop codon sequence insertion. **(B)** Western blot assay to confirm TcZC3HTTP gene disruption. TcZC3HTTP Hemi-knockout cloned population were transfected once again with gRNAs targeting TcZC3HTTP (gRNA 99 or gRNA 231) or a non-guide RNA control (Ctrl) and parasites were harvested and protein content extracted. Polyclonal antibodies against TcZC3HTTP (1:1000) were used for protein detection. The amount of protein extract applied corresponded to 1 × 107 parasites and TcAKR protein was used as an input control. This experiment was performed twice.

In order to further explore the hemi-knockout hypothesis, the clones that presented the *Bgl*II restriction site but also showed undigested product (meaning that they were not null mutants) were single cell cloned once more and further analyzed by PCR following a *Bgl*II digestion. All clones tested were partially digested with *Bgl*II (data not shown), indicating that they were in fact hemi-knockouts. Thus, at least two hemi-knockout clones for each ZFP target gene were submitted to a new transfection round with the corresponding gRNA and DNA donor previously used. This approach allowed to confirm the knockout of TcZC3HTTP gene by detecting the DNA donor insertion and complete PCR product *Bgl*II digestion and also loss of TcZC3HTTP protein expression by Western blot (Figure 6). We keep searching for null mutant clones to TcZC3H39 and TcZC3H29 coding genes as we will advance with the TcZC3HTTP knockout parasites to the next step, that is to further investigate its related phenotype, specially regarding its impact in the context of *T. cruzi* gene expression regulation.

## Discussion

RBPs are considered essential factors in gene expression regulation, especially in trypanosomatids, where posttranscriptional processes are predominant. Indeed, advances in the understanding of RBP functions reinforce their actions as key players in coordinating mRNA metabolism and maintaining cell homeostasis. However, the contributions of several RBPs to the *T. cruzi* regulatory network remain to be determined. From all the RBPs studied in *T. cruzi* presented in this review, until now, only the function of TcZC3H31 has been investigated by a gene knockout approach (Alcantara et al., 2018). This lack of studies regarding RBP knockout in *T. cruzi* is mainly due to the challenge of genetically manipulating this parasite using classical knockout approaches, which are limited in the case of essential genes. In this context, the development and adaptation of the genetic editing tool, CRISPR/Cas9, has emerged as a great alternative to achieve RBP knockout in *T. cruzi*.

As a proof of concept, along with this review we present data from the knockout of three RBPs with C3H zinc finger domains in *T. cruzi* by using the CRISPR/Cas9 technique. TcZC3H39, TcZC3H29, and TcZC3HTTP knockouts were achieved using two distinct approaches. One consisted of transfecting specific gRNAs for each target gene only into a Cas9-GFP-expressing population. The other approach involved co-transfecting a specific DNA donor template along with the related gRNA. By targeting the tczc3h39, tczc3h29, or tczc3http gene, we were able to see major morphological changes, cell cycle impairment (Figures 3, 4, respectively) and viability loss (data not shown). There were characteristic morphological alterations for each culture, and all modifications involved an increase in cell size and in the number and/or shape of the flagellum (Figure 3). Burle-Caldas and colleagues reported that disrupting GP72 without a DNA donor sequence to direct repair could lead to abnormal morphology depending on the gRNA used (Burle-Caldas et al., 2018). Although the changes observed in the knockout cultures for TcZC3H39, TcZC3H29, and TcZC3HTTP were not seen in the controls (RNA control, gRNA-GFP, Cas9-GFP transfected with PBS), or even with the targeting of other non-RBP genes [e.g., GP72, α-tubulin, β-tubulin (Romagnoli et al., 2018)], this raises a question about the specificity of the phenotypes observed for the zinc finger gene knockouts. However, it is worth mentioning that morphological changes similar to those we observed for TcZC3H39 and TcZC3H29 knockouts were also described when overexpressing TbZFP3 (Paterou et al., 2006) or when knocking down the RBPs ALBA3/4 (Subota et al., 2011), TbRRM1 (Levy et al., 2015), and TbZFP2 (Hendriks et al., 2001) in *T. brucei*. In 2001, Hendriks et al. identified a posterior end elongation phenotype in TbZFP2 knockdown parasites (caused by the polar extension of microtubules) that they termed a “nozzle” (Hendriks et al., 2001). Interestingly, TcZC3H39 and TcZC3H29 knockout parasites displayed a morphological phenotype resembling the “nozzle.” Moreover, the G2/M phase cell cycle arrest observed in the zinc finger RBP knockouts described herein (Figure 4) was also described in *T. brucei* when suppressing the genes encoding the RBPs TbPUF9 (Archer et al., 2009), ALBA3/4 (Subota et al., 2011), TbZC3H11 (Droll et al., 2013), TbRRM1 (Levy et al., 2015), and TbZFP2 (Hendriks et al., 2001) by RNAi. These data corroborate the notion that the cell cycle alterations identified in the knockout populations for TcZC3H39, TcZC3H29, and TcZC3HTTP are specific and not the result of the non-specific activity of endonuclease Cas9. In addition to all the controls used in our experiment, the phenotypes reported in *T. brucei* came from RNAi studies and, therefore, did not involve DNA editing (double-strand breaks). When the expression levels of the aforementioned *T. brucei* RBPs were suppressed/abolished, there were effects on the cell cycle accompanied by major morphological changes, such as the nozzle phenotype. Hence, there is a strong correlation between cell cycle and cell morphology maintenance with RBPs. It remains to be elucidated whether this is a direct or indirect relation (through their RNA targets). Either way, this is the first evidence showing the nozzle phenotype and relating these morphological changes with cell cycle arrest due to the knockout of zinc finger proteins in *T. cruzi*.

To further support the idea that the observed phenotypes are specific to TcZC3H39, TcZC3H29, and TcZC3HTTP knockout, the co-transfection of DNA donor along with gRNAs allows detection of the precise insertion of the repair template into the target genes (Figure 5). However, although the previously described phenotypes (without DNA donor transfection) were reproduced, they were significantly less frequent relative to the approach using only gRNAs (Supplementary Figure 6). We found evidence that this was due to the selection of hemi-knockout populations (Figure 5D). How unintentional heterozygote parasites are generated by CRISPR/Cas9 remains to be explained, but it seems to occur preferably when cells are co-transfected with the DNA donor. Curiously, another group working with *T. cruzi* also observed this phenomenon (Soares Medeiros et al., 2017). In their report, Soares Medeiros et al. (2017) attempted to knock out the Galf and calreticulin (CRT) genes by CRISPR/Cas9 but only found clones presenting both WT and mutant alleles. Since those genes are single-copy genes and, for CRT, knockout by conventional approaches was unfruitful, authors associated the heterozygosity to genes that are potentially essential to the parasite (Soares Medeiros et al., 2017). According to this and based on all the evidence gathered from the phenotypes related to the disruption of TcZC3H39, TcZC3H29, and TcZC3HTTP, we believed that the generation of a hemi-knockout population reinforces the idea that these zinc finger genes may be essential for *T. cruzi*.

Performing a new round of transfection in those hemi-knockout populations allowed us to obtain null mutant clones for TcZC3HTTP, thus indicating that this protein is not essential in epimastigotes. However, since TcZC3HTTP is downregulated throughout metacyclogenesis, a lethal phenotype could be observed in this differentiation process. Therefore, it is very important to characterize these clones during this development stage.

The use of the CRISPR/Cas9 technique for RBP knockout will start to open a new era in gene expression regulation studies and advance the uncovering and mapping of regulatory gene networks in *T. cruzi*.

## Materials and Methods

### *T. cruzi* Culture, gRNA and DNA Donor Preparation and Transfections

*T. cruzi* Dm28c epimastigotes were cultured at 28°C in liver infusion tryptose (LIT) medium supplemented with 10% heat-inactivated fetal bovine serum (FBS). The guide RNAs were obtained using the online Eukaryotic Pathogen CRISPR gRNA Design Tool (EuPaGDT) (Peng and Tarleton, 2015) and produced by *in vitro* transcription as previously described (Peng et al., 2015; Romagnoli et al., 2018). DNA donor sequences were designed to have a *Bgl*II restriction site (AGATCT) and a sequence encoding stop codons in three different frames (TAGATAGATAG), all flanked by 30-nt homology arms (according to their specific gRNA target sequence). gRNAs and donor sequences are in Supplementary Table 1. For transfection, 5 × 10^6^ early-log phase Cas9-GFP expressing epimastigotes were harvested by centrifugation (3,000 × g, 5 min), washed in PBS (pH 7.4) and resuspended in 100 μl of human T cell nucleofector solution at room temperature. For target gene disruption, 20 μg of a specific gRNA and 20 μg of respective donor DNA were added to the solution before electroporating the parasites with one electric pulse using the X-014 program in an Amaxa Nucleofector device. A DNA fragment of human 18S rRNA provided by the MEGAShortscript T7 kit (Thermo Fisher Scientific) was transcribed and used as a control (RNA control). After transfection, parasites were cultured in 25-cm^2^ cell culture flasks containing 10 ml of LIT medium supplemented with 10% FBS.

### Immunolocalization and Immunoblot Assays

For immunofluorescence assays, epimastigotes were harvested from culture, washed, fixed with 4% paraformaldehyde in PBS and added to poly-L-lysine-coated slides. Cells were then permeabilized with Triton X-100 in PBS (pH 8.0) for 5 min and blocked with bovine serum albumin (BSA, 1.5%). Parasites were incubated with anti-TcZC3H29 (1:300) or anti-TcZC3HTTP (1:750), and an Alexa 488-conjugated goat anti-mouse antibody (1:600) was used for detection. The nuclei and kinetoplast were stained with DAPI. Images were collected on Leica DMI6000 B (Leica-microsystems) equipment. Captured images were processed by deconvolution with LAS AF–Leica (Leica microsystems) software. Bar = 10 μm.

For western blot assays, wild-type and transfected epimastigotes (expressing TcZC3H29-3xFLAG or TCZC3HTTP-3xFLAG) were differentiated into metacyclic trypomastigotes as previously described (Contreras et al., 1985), and protein extract from different stages during differentiation was prepared. Protein extracts were separated by SDS-PAGE (13%) and transferred to a nitrocellulose membrane. After Ponceau S staining and blocking non-specific binding sites with 5% non-fat skim milk in PBST (PBS supplemented with 0.05% of Tween 20) for 1 h at 25°C, membranes were incubated with anti-TcZC3H29 (polyclonal, 1:500), anti-TcZC3HTTP (polyclonal, 1:1000), anti-TcAKR (polyclonal, 1:1000) or anti-FLAG antibodies (monoclonal, 1:1000) for 1 h at 25°C, washed three times with PBST and incubated with goat anti-mouse IgG secondary antibodies conjugated to the fluorophore IRDye®680LT (LI-COR Biosciences). Fluorescence detection was performed with an Odyssey® scanner (LI-COR Biosciences).

### Morphological Characterization

Panoptic staining and sample preparation and acquisition for scanning electron microscopy were performed as previously described (Romagnoli et al., 2018).

### Flow Cytometry

DNA content determination was performed in a FACSCanto II machine (Becton-Dickinson). A total of 1 × 10^6^ parasites were harvested (3,000 × g, 5 min) and resuspended in 100 μl of PBS and mixed with 100 μl propidium iodide (PI) staining solution (3.4 mM Tris-HCl pH 7.4, 0.1% NP40, 10 μg/ml RNAse A, 10 mM NaCl, 30 μg/ml propidium iodide). PI was excited by a blue laser (488 nm), and emitted light was collected by 585/42 bandpass. Single cells were gated based on pulse area (PE-A) vs. pulse width (PE-W) of the PE channel. Cellular aggregates and debris were excluded from cell cycle analysis. At least 10,000 events were recorded for each replicate, and data were analyzed using FlowJo V10.1r7 software. For single-cell sorting and cell enrichment, a BD FACSARIA II (Becton-Dickinson) machine was used.

### DNA Amplification, Digestion, and Sequencing

DNA amplification was carried out by PCR with specific primers for TcZC3H39 (TCDM_00519), TcZC3H29 (TCDM_11529), and TcZC3HTTP (TCDM _03704) (Supplementary Table 1) directly from liquid culture as previously described (Alcantara et al., 2014). PCR conditions were as follows: 95°C for 2 min, followed by 40 cycles of 95°C for 15 s, 55°C for 15 s, and 72°C for 1 min and 15 s. Additionally, for pGEM-T easy (Promega) amplicon cloning, a final step of 72°C for 10 min was included. PCR products were digested by *Bgl*II (New England Biolabs) at 37°C overnight. Amplified DNA and digestion products were visualized in a 3% agarose gel. For sequencing, plasmids containing the disrupted genes (previously confirmed by PCR and digestion) were purified with the QIAprep Spin Miniprep Kit (QIAGEN) and sequenced at WEMSeq Biotechnology (wemseq.com). Alignments were made using MEGA version 10.0.5.

## Data Availability Statement

The raw data supporting the conclusions of this article will be made available by the authors, without undue reservation, to any qualified researcher.

## Author Contributions

BR performed the experiments and wrote the manuscript. FH discussed the results and wrote the manuscript. LA collaborated on the discussion of the results and wrote the manuscript. SG collaborated on the discussion of the results and wrote the manuscript. All authors approve the submitted version.

### Conflict of Interest

The authors declare that the research was conducted in the absence of any commercial or financial relationships that could be construed as a potential conflict of interest.
