# Supplementary material for: RNA Binding Proteins and Gene Expression Regulation in Trypanosoma cruzi
Source: Front Cell Infect Microbiol. 2020 Feb 20;10:56. doi: 10.3389/fcimb.2020.00056 (PMC7045066; doi:10.3389/fcimb.2020.00056)
Supplement: Supplementary file 2 [file Data_Sheet_1.DOCX]

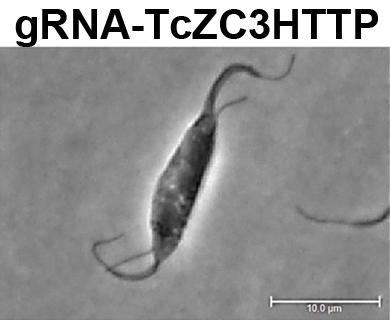


**Supplementary Figure 1. Targeting TcZC3HTTP affects cell division coordination.** After 3 days post transfection with gRNAs targeting TcZC3HTTP, parasites at final stages of cell division were seen with two flagella at each end.


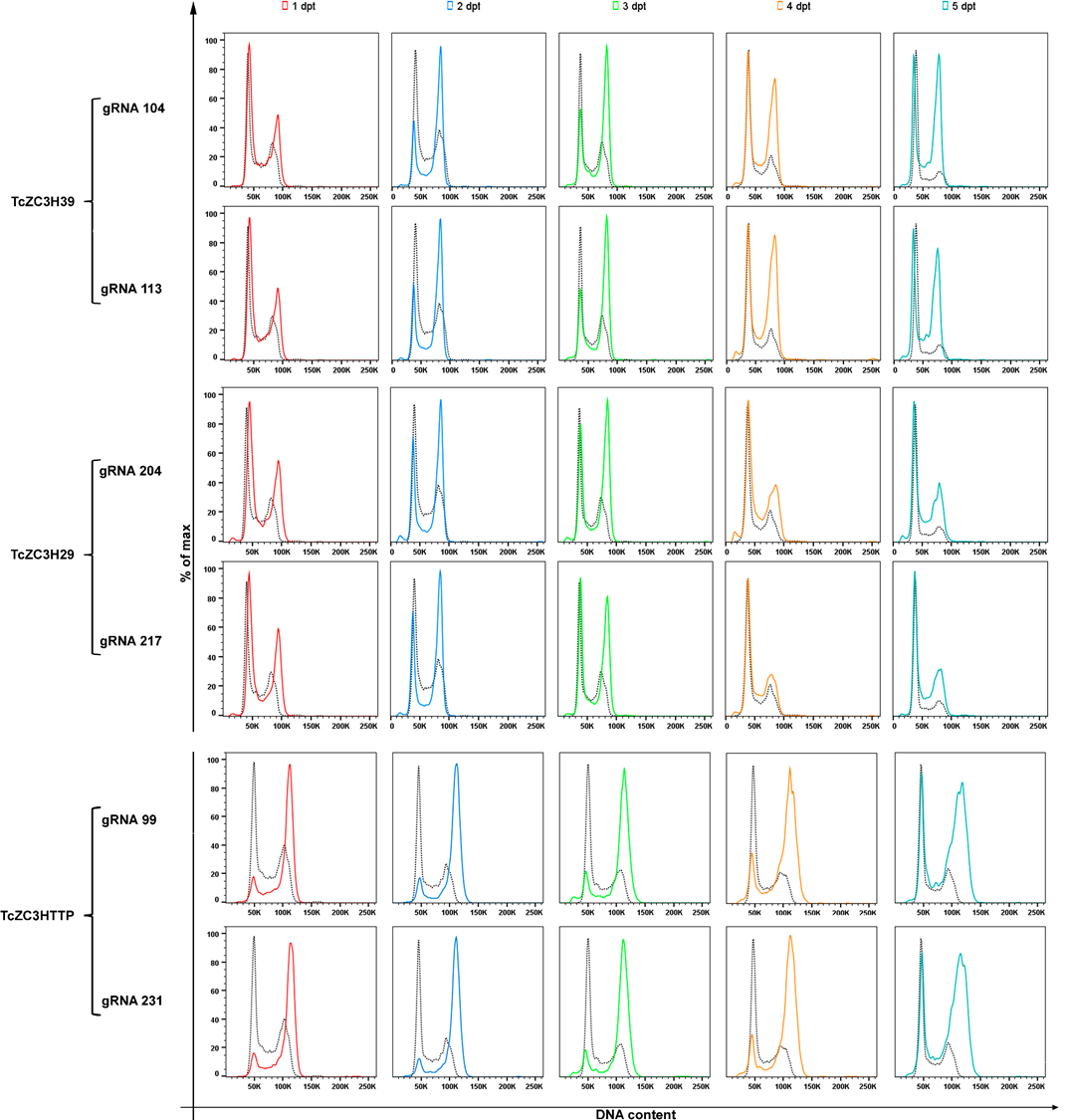


S**upplementary Figure 2. Cell cycle impairment in cultures transfected with gRNAs targeting tczc3h39, tczc3h29 or tcz3http.** Cas9-GFP expressing parasites were transfected with guide RNAs targeting TcZC3H39, TcZC3H29 or TcZC3HTTP encoding genes and had their DNA content assessed by flow cytometry. Graphs show percentage of total cells (y axis) by the amount of DNA (x axis) at each day (columns) in comparison to the wild-type population (black dotted). Each row presents data obtained from one gRNA as two different guides were used to edit each ZFP target gene.


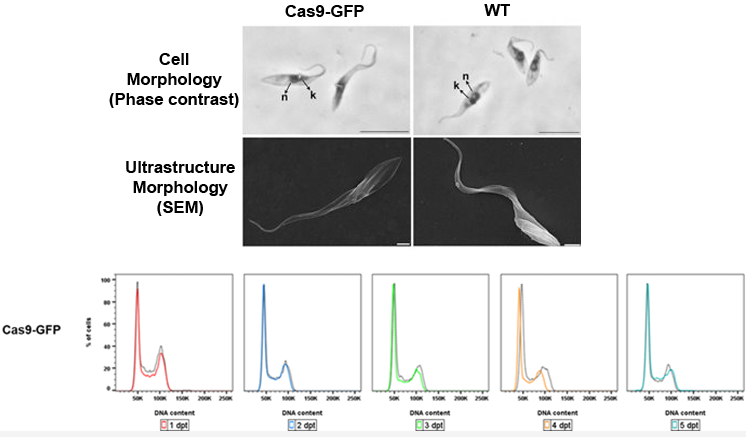


**Supplementary Figure 3. Cell cycle and morphology of transfected control cultures.** Cas9-GFP expressing (Cas9-GFP) and wild-type parasites (WT) were visualized by light microscopy (upper lane) and scanning electron microscopy (bottom lane) after transfection with PBS (instead of gRNAs). The nucleus (n) and kinetoplast (k) are indicated. Bar: 10 µm (light microscopy) and 2 µm (SEM). Cell cycle analysis revealed that transfected Cas9-GFP expressing parasites (without gRNAs) had no impact on their cell cycle during the whole experiment (from day 1 to 5 after transfection) Graphs show percentage of total cells (y axis) by the amount of DNA (x axis) in each day (columns) in comparison to the wild-type population (black dotted).

**
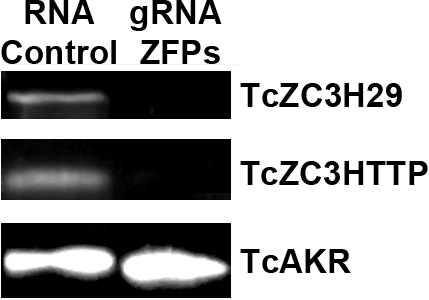
**

**Supplementary Figure 4. TcZC3H29 and TcZC3HTTP knockout confirmation after transfection with specific gRNAs.** Western blot assay to confirm target genes disruption. Populations were transfected with a specific gRNA to TcZC3H29 or TcZC3HTTP (gRNA ZFPs) or a non-guide RNA control (RNA ctrl) and, in the third day posttransfection, parasites were harvested and protein content extracted. Polyclonal antibodies against TcZC3H29 (1:500) or TcZC3HTTP (1:1000) were used for protein detection. In both lanes the amount of protein extract applied corresponded to 2 x 10^7^ parasites. The TcAKR protein was used as an input control.


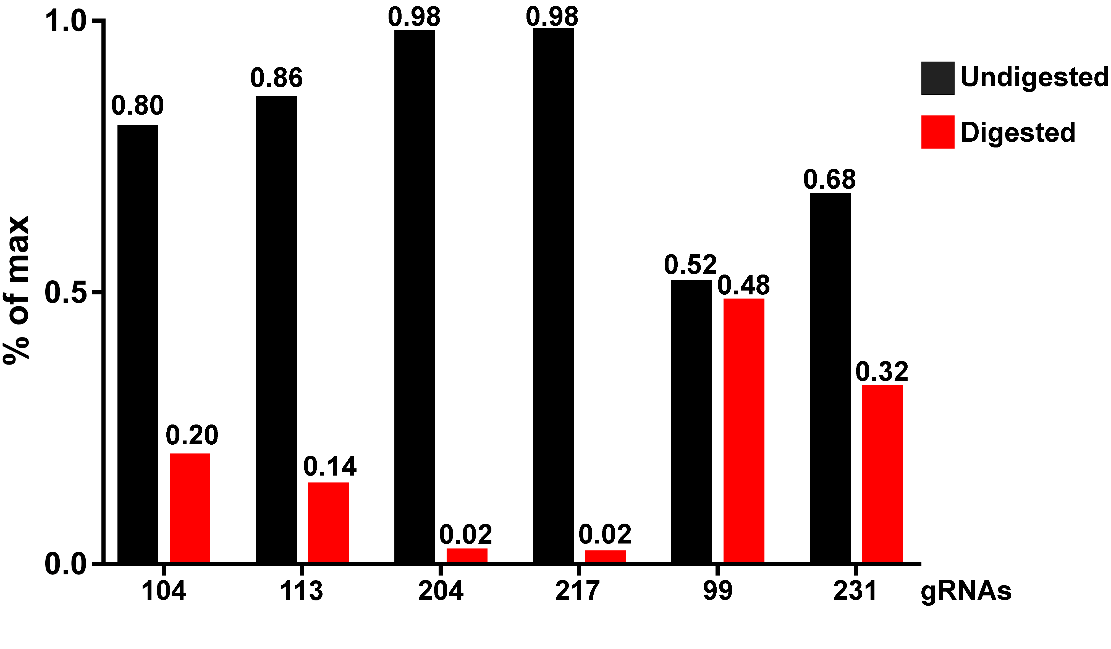


**Supplementary Figure 5. Band intensity quantification to measure % of DNA donor incorporation.** Digested and undigested bands were identified and quantified using the ImageJ software v1.52a. The intensities of each lane (digested or undigested PCR product from a transfected culture) were calculated based on the overall intensity for that lane (digested + undigested signal) and then individual signals were calculated proportionally (% are expressed above each corresponding bar). Graphs were plotted with the Graphpad Prism 7 software.


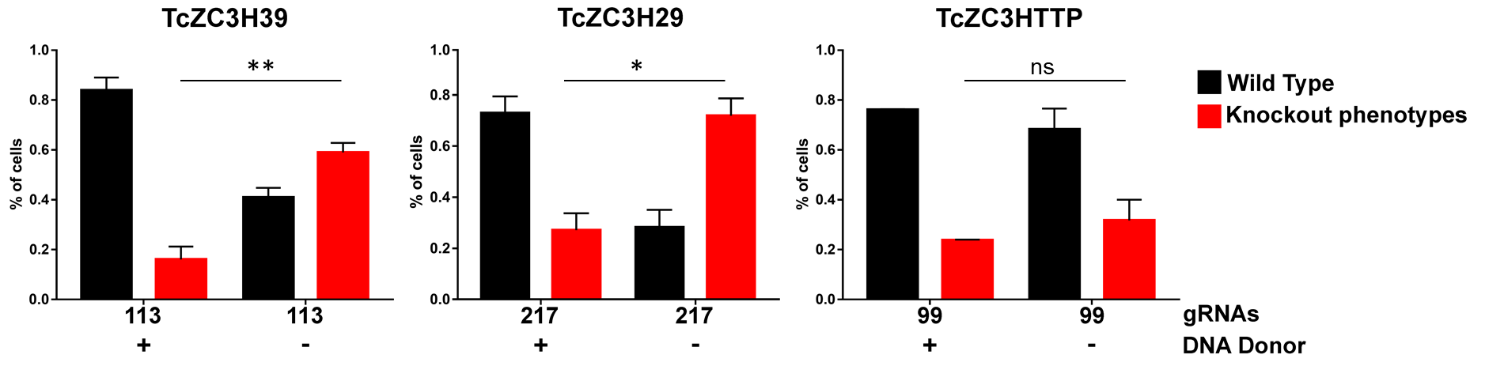


**Supplementary Figure 6. Comparison of knockout phenotypes frequencies in populations transfected with specific gRNAs targeting TcZC3H39, TcZC3H29 or TcZC3HTTP with (+) or without (-) the related DNA donor.** Three days post transfection parasites were harvested, washed, fixed with 4% paraformaldehyde in PBS and added to poly-L-lysine-coated slides. For phenotype quantification, images were taken from distinct fields and parasites were classified according their morphology. At least a hundred parasites were counted in each condition (with or without the DNA donor). Graphs were plotted and data analyzed with the Graphpad Prism 7 software. Statistical analysis was performed using 2way ANOVA and Tukey’s multiple comparisons test. N=2; * p<0.05; ** p<0.01; ns – not significant)
